# Conceptual framework of the nursing centre for the integration of community health nursing practice, education, and research

**DOI:** 10.1017/S1463423619000331

**Published:** 2019-07-01

**Authors:** Neti Juniarti, Jeffrey Fuller, Lana Zannettino, Julian Grant

**Affiliations:** 1 Community Health Nursing Department, Faculty of Nursing, Universitas Padjadjaran, Indonesia, Jl. Raya Bandung – Sumedang KM. 21 Jatinangor, West Java, Indonesia; 2 College of Nursing and Health Sciences, Flinders University of South Australia, Sturt Road, Bedford Park 5042, South Australia, Australia

**Keywords:** case study, community health nursing, conceptual framework, Nursing Centres, nursing education

## Abstract

**Aim:**

To develop a conceptual framework that can be used for the integration of community health nursing (CHN) practice, education, and research within a Nursing Centre (NC) model.

**Background:**

New forms of training and support are needed to equip nurses to manage the complex and costly challenges facing health care systems. The NC model provides scope to address these challenges by integrating nursing practice, education, and research. However, there is little information about how these constructs are integrated or how education is constituted within the model.

**Methods:**

This study used an embedded single case study design across three Nursing Centres (NCs) in West Java Indonesia. Semi-structured interviews and a review of relevant documents were conducted. Interview participants were recruited purposively to select stakeholders with rich information, including clients, nurses, nursing students and lecturers who have been using the NC model, as well as the head of the co-located Community Health Centres. Data was analysed using thematic analysis, pattern matching and cross-unit synthesis.

**Findings:**

Four components relevant to integration in the NC were identified, namely (1) client-centred care as the shared common ground for integration in the NC; (2) nursing education using a service learning approach; (3) the NC as a model for reviving CHN services; and (4) service improvement through research and community service activities. The service learning approach was identified as appropriate because it links services with the learning process and this serves to address the interests of both practice and education institutions. The conceptual framework identified in this study can be used to improve the functionality of NCs in Indonesia and be considered for use internationally.

## Introduction

New forms of training and support are needed to equip nurses to manage the complex and costly challenges facing health care systems. To provide appropriate forms of training and support, strong partnerships through organisational integration are needed between nursing schools and health care organisations (Wilcock, 2011, p. 242). Inter-organisational integration refers to the quality of collaboration among different organisations and different sectors in the society to improve the quality of service as well as to improve efficiency (Axelsson and Axelsson, 2006). This can also extend to the way in which services are delivered and how practices are organised and managed together (Heath *et al*., 2013). The main types of inter-organisational integration are co-operation and collaboration, although terms such as networks, partnerships, and coalitions are also used to explain different levels of integration (Axelsson and Axelsson, 2006). Cooperation and collaboration is a continuous process at both individual and system levels where individuals are required to constantly negotiate boundaries (Wihlman *et al*., 2008, p. 1).

Integration of academic and health care organisations is often difficult to establish due to lack of trust and respect from both parties (Andrew *et al*., 2008). This can result in gaps between what is taught and what is practised, presenting major challenges for nursing as a profession (Kitson, 2006; Maben *et al*., 2006). Nursing education can then have little immediate or direct impact on achieving goals of quality patient care (Smith and McCarthy, 2010). Proposed solutions include integrating academic research in practice settings to enhance the implementation of evidence-based practice throughout nursing (Christie *et al*., 2012).

Schools of Nursing in the USA established Nursing Centres (NCs) in the previous decade as one approach to bridging the gap between education and practice (King, 2008). These centres have a threefold goal to educate student nurses, provide health care services and conduct research that advances health care (Pohl *et al*., 2007). As a part of community health care and higher education systems, NCs sought to integrate scholarly nursing practice, education and research to provide a comprehensive primary health care service to individuals, families and communities (Boettcher, 1996; Shiber and D’Lugoff, 2002; Barkauskas *et al*., 2006). In the USA, the origin of the Nursing centre (NC) was started in the late 19th century (King, 2008). The NCs were defined as:

*Organisations that give clients and communities direct access to professional nursing services. Professional nurses in these centres diagnose and treat human responses to actual and potential health problems, and promote health and optimal functioning among target populations and communities (American Nurses’ Association, 1995, p.1).*

There are two major approaches to the NC. These include the approach that focusses on integration between nursing and other organisations (Lundeen, 1993; 1999) and that focusses on integration of health services, research and education (Newman, 2005). While most of these papers reported on the integration of nursing practice, education and research, there was little information about the integration frameworks or specific approaches to education. Miller *et al*. (2004) proposed that a business plan as a blueprint for the NC should be used to determine the feasibility of clinical services, faculty development requirements, and expected returns on investment of time and resources. However, there has thus far been no subsequent publication that indicates that this blueprint has been implemented and evaluated.

In West Java, Indonesia, an NC model was developed in 2002 by the Faculty of Nursing *Universitas Padjadjaran* (UNPAD) (Samba, 2002). Defined as a nurse-led clinic, the model integrates health care services, education and research through the optimal use of all potential resources in the community health care system (Samba, 2007). The NC model in West Java is unique because attachment to the government-owned community health centres is the main integration strategy. It emphasises improving the quality of Community Health Nursing (CHN) services, education, and health outcomes for people in the community. This NC was the first collaborative project between nursing education institutions, local government, provincial health offices, community health centres (*Puskesmas*) and the community in Indonesia. In 2016, there were 26 NCs located in 21 cities and regencies in West Java Province. By 2018, this has increased to 36 NCs.

One of the problems for NCs around the world is sustainability (Barger and Kline, 1993; Bell, 2008). Thus, organisational partnership between academics and the community is needed to maintain the sustainability of NCs (Krothe *et al*., 2000). However, there is a lack of a conceptual framework from which to compare what works and the benefits of partnership for stakeholders (Levine-Brill *et al*., 2009; Funnel and Rogers, 2011; Leffers and Mitchell, 2011). Also, there are no clear indicators of success and there is also a lack of a blueprint that can be used to measure the effectiveness of the model (Levine-Brill *et al*., 2009). A lack of clear measurement indicators is quite common in organisations that have a diversity of practice and a wide variety of stakeholders (Davies, 2004). As the NC model is increasing in use in Indonesia, it is essential that frameworks are established to measure their success and identify areas for improvement. This paper presents research that identified the core components of the NC in West Java, and proposes a conceptual framework that can be used for the research and evaluation of NCs in Indonesia.

## Methods

This study used an embedded single case study design (Gomm *et al*., 2000; Yin, 2014). Guided by a constructivist ontology and an interpretivist epistemology, this study used an embedded single case study design as the strategy to conduct the research (Yin, 2014). This study used a case study design to investigate the activities and processes of, and the inter-relationships between, the stakeholders in the NC model within its real-life context in Community Health Centres *(Puskesmas)* in West Java, Indonesia. The findings of this study have informed the process of developing a theory to underpin the NC model.

The model is viewed as the single case because it is a unique and collaborative CHN educational approach in the Indonesian setting. While the NC model is viewed as a single case, the study involved three NC (Puskesmas) sites in West Java as the embedded sub-units of analysis. These sub-units were used as focussed sites for the case study inquiry to obtain sufficient and specific data about the NC model as the larger unit of analysis (Yin, 2014). Even though case study findings cannot be used for empirical generalisation, the wider relevance of the findings may be conceptualised as a basis of transferability to other settings (Gomm *et al*., 2000; Yin, 2014). The study was approved by the Flinders University of South Australia’s Social and Behavioural Research Ethics Committee (SBREC) (project number 5887).

### Data collection

Data included semi-structured interviews with a range of stakeholders (*n* = 41) and a search of evidentiary materials that comprised the original documents about the NC model, two published books about NCs, and policies related to nursing education, CHN practice, and community health centres in Indonesia (*n* = 9).

The focus of the study was on the experiences of clients, the nurses, nursing students, and lecturers who have been using the NC, as well as the head of the Community Health Centre. Hence, participants were recruited purposively in this study in order to select cases and stakeholders who could provide rich information for the in-depth study of participants’ opinions, interpretations, and perspectives (Liamputtong and Ezzy, 2005). Participants included clients, nurses, nursing students and lecturers using the NC model, as well as the head of the co-located Community Health Centres (*Puskesmas*). The number of participants recruited from each of the three sites was 13. As suggested by Guest *et al*. (2006), at least twelve interviews per site is needed to understand common perceptions and experiences. With the founder of the NC and the Provincial Coordinator, the total number of participants was 41. The characteristics of participants who were involved in this study are presented in Table 1.

**Table 1.** Participants characteristics

Characteristics of participants		NC 1	NC 2	NC 3	Total
Type	Students	3	3	3	9
	Lecturers	3	3	3	9
	Nurses	3	3	3	9
	Clients	3	3	3	9
	Head of *Puskesmas* who is also a physician		1	1	2
	Head of Puskesmas who is not a physician	1	–	–	1
	The founder of the NC in West Java	–	–	–	1
	Provincial coordinator of CHN program	–	–	–	1
Sex	Male	3	1	3	7
	Female	10	12	10	32

The semi-structured interviews comprised open-ended questions that encouraged participants to express their perceptions about the NC in a conversational manner, while closely maintaining the case study line of inquiry (Yin, 2014). All the interviews were conducted in the Indonesian language by the first author who is an Indonesian native speaker. Interviews ranged from 30 minutes to 1.5 hours in duration and were recorded using a digital audio recorder. Field notes were also taken by the interviewer during the interviews.

The questions asked during the semi-structured interviews were informed by a study conducted by Laplante (2009) which investigated the meaning of reciprocity in service learning, and also by the Kellogg logic model which consists of examining context, implementation, and outcomes (Kellogg Foundation, 2004). The interview questions focussed on the context of the NC model, the activities and implementation of this model, and the perceived outcomes of the NC. Participants were also asked about their understandings of the learning methods involved in the NC model. The matrix of interview questions is presented in Table 2.

**Table 2.** The matrix of interview questions for seven stakeholders

Objective	Key information/indicators	Interview schedule	Client	Nursing students	Nurses	The founder of the NC	Coordinator of provincial public health nursing program	The head of public health centres	Lecturers
To identify the theoretical basis and purpose in the establishment of the NC.	The reason in establishing the NC model	Why do you initiate the NC model at the first place?				V	V		
	Concepts or theories for the NC model	What are the concepts underlying the establishment of the NC?				V	V	V	V
	Public health nursing practice in Public Health Centre (Puskesmas)	Compared to Puskesmas without the NC, do you think the nurses’ involvement in the NC will assist them in their future career? – please explain			V		V	V	
	Nursing education practice in the NC	Compared to usual clinical placements, do you think that students’ involvement in the Nursing Centre will assist them in their future career (such as in terms of knowledge and skills) – please explain?		V	V	V	V	V	V
		Compared to usual clinical placements, do you think that lecturers’ involvement in the Nursing Centre will assist them in their future career (such as in terms of knowledge and skills) – please explain?				V	V		V
To describe the policy and resources context that has influenced the establishment of the NC	Policy of the public health nursing in Indonesia	What is the role of provincial health office and district health office to the development of the NC?					V	V	
		Why provincial and district health office support the establishment of the NC?					V		
	Policy of establishment of the Nursing Centre	What is the policy from provincial health office and Ministry of Health regarding the NC?					V		
	Resources that influencing the establishment and operationalisation of the NC model	What are the factors (internal or external) that influence the establishment and operationalisation of the NC model?				V	V	V	
To describe the implementation of three NCs within existing public health centres in West Java.	The process and implementation of the NC model	What are the factors that influence the process and implementation of the NC model?	V	V	V			V	V
		Based on your experience, are there any NC that is not working well? If yes, why do you think this happen?							
		How is the relationship between nurses and students in the NC?							
To explore the stakeholders’ perception of the NC as a model of service learning.	Perceptions of consumer	What do you think are the strengths and weaknesses of the Nursing Centre, to nursing students, to the nurses who work in the Centre, to the community? Do you have suggestion about how to improve the Nursing Centre model in the future? Is there anything else that you would like to say about the Nursing Centre?	V	V	V	V	V	V	V
	Perceptions of care providers		V	V	V	V	V	V	V
	Perceptions of managers		V	V	V	V	V	V	V
	Perceptions of other stakeholders		V	V	V	V	V	V	V
To develop a program theory model that will enable the evaluation of the NC as a model of service learning in community health in Indonesia	Based on all the interview data above.	What is the design for evaluating the NC, and who is doing the evaluation? What are the outcome measures being used, and what outcomes have been identified to date? What rival explanations have been identified and explored?			V	V	V	V	V

### Data analysis

The audio recordings of the interviews were transcribed in the Indonesian language, forward-translated into English, and then back-translated into Indonesian to ensure the rigour of the qualitative data. All authors were involved in identifying and refining the themes of three interviews transcripts in order to ensure that the coding process was comprehensive and to identify the ‘scope’ of what each theme was about. The first author continued the analysis using the same coding process for all interview transcripts. NVIVO version 10 software (QSR International Pty Ltd., 2012) was used to store and organise the data. Data analysis was undertaken in two stages. Firstly, inductive thematic analysis was used to identify the experiential issues described by the participants (Braun and Clark, 2012). The analysis consisted of the following six phases: becoming familiar with the data, generating the initial codes, searching for themes, reviewing the themes, defining and naming the themes, and producing the report. Pattern matching and cross-case synthesis (Yin, 2014) were then employed as a second stage to conceptualise the broad components of the various puskesmas activities, informing the overall NC model. The identification of patterns and consistency in the case study approach is considered the most rigorous technique for case study researchers (Yin, 2014). This is because the identified patterns can show how the various parts of the findings fit together (Thomas, 2011). The researcher compares the pattern of the data empirically based on the expected pattern of outcomes (Yin, 2014). Pattern matching enabled comparison of study findings with the theoretically expected outcomes from the literature and evidentiary materials in order to examine the most suitable educational method to be used in the NC model (Thomas, 2011). The pattern of relationships found in the data from the three NCs was then compared to the pattern of possible explanations using the components of adult learning, active learning, and service learning theories, as these are the concepts that are closely related to the NC model in Indonesia. In this way, the findings of this study will reflect the most suitable educational method to be used in, and to improve, the NC model. If the empirical pattern and the possible explanation seem to be congruent, the findings can strengthen the credibility of the study (Yin, 2014).

The cross-unit synthesis was used to compare and contrast the three NC sites to draw a broad conclusion about the ideal NC model. Themes were then grouped to identify the similarities and differences in the situation, implementation and outcomes of the NC across the three sites.

### Rigour

To be considered as evidence, a qualitative narrative should consider the rigour or trustworthiness of the interpretive perspective based on its credibility, transferability, confirmability, and dependability (Patton, 2002; Altheide and Johnson, 2011). Creswell (2009) suggested the use of triangulation as one way to maintain the credibility and accuracy of the findings. In this study, methodological triangulation was employed by using more than one data collection technique; that is semi-structured interviews and document analysis. Data triangulation was also achieved by using multiple data sources from seven NC stakeholders. Documents were also used as additional sources of evidence. The documents were analysed to identify the original Indonesian NC model, the development of the NC model, the specific activities expected to be conducted by the NC, the history and development of policy for CHN practice in Indonesia, and to understand the underlying reasons for collaboration between nursing education institutions and community health centres.

In terms of transferability, qualitative research seeks to understand, in an in-depth manner, a specific case within a particular context, rather than to generalise. In this study, the NC stakeholders have differing views which need to be explored in order to deeply understand them. Even though these views could not be generalised, the lessons learned from a deep understanding of the stakeholders’ views can be used to inform other models beyond the case study site of investigation (Yin, 2014).

To ensure the dependability of this research, steps of analysis were explained, starting from methods, data collection procedures, and data analysis procedures. In terms of confirmability, authors were constantly aware of the possibility of bias in the data collection. Authors worked within the reflexive approach constantly in order to consciously separate personal background from the data, and to analyse the data using an ‘outsider’ perspective. In this way, the personal bias was kept to minimal

## Findings

### The functionality of the Indonesian NCs

Overall, the current condition of the three NCs demonstrates that there is an inadequate functionality of the NCs because the components of the NC model have been understood differently by the various stakeholders. Each of these centres has a different focus, with NC 1 focusing on student placement for CHN and family nursing, for example ‘*The operation of the NC was mostly conducted by the students. […] So far in here, the NC was a place for students to learn to provide nursing services*’ (Lecturer 1 NC1). NC 2 focusing on lecturers’ community service activities, ‘*the NC is used by the lecturers only for community service. We don’t involve students in the NC*’ (Lecturer 1 NC2). NC 3 focusing more on the Community Health Centre agendas that leads to the ineffective nursing education in this NC, as stated by one of the students:

*I have done a placement in the community and have heard about the NC from the lecture. However, placement in the NC is not effective because we [male students] have not been given a chance to practice inside the Puskesmas (Student 1 NC3)*.

As a result of these three foci, the optimal collaboration between services and learning experience in the NCs was not able to be developed, implemented, monitored or evaluated. In order to improve the integration and hence the functionality of the NC, the data were analysed to identify the ideal components of the NC as perceived by the participants. These components are presented in the following sections.

### Main components of the NC

Four main components of the NC were identified as: (1) client-centred care as the shared common ground for integration in the NC; (2) nursing education using a service learning approach; (3) the NC as a model for reviving CHN services; and (4) service improvement through research and community service activities. It is argued that all of these components need to operate in a conducive environment such as the feeling of being valued in order to develop the functionality of the NC.

### Client-centred care as the shared common ground for integration in the NC

To improve the functionality of the NC, the one basis for common ground identified in this study was to focus on clients, including individuals, families, and people in the community within a caring environment. Most of the stakeholders viewed that the NC has benefitted clients. The following excerpts are examples of students’ and nurses’ views about this:

*The benefit of the NC is very big for people in the community because they can receive free health consultations. People do not have to spend money, but they will know how to manage their illness, such as TB and diabetes mellitus (Student 3 NC1)*.

*Patients can receive more knowledge in the NC, we can prevent patients from getting worse, […] we explain how to take care of the disease at home so that the patients can be independent in conducting self-care (Nurse 1 NC3)*.

Client-centred care is the core requirement that could be used as a starting point and a common ground towards the improvement of health outcomes for individuals, families and people in the community through close collaboration between the nursing education institution and the *Puskesmas*. This client-centred appraoch had helped to develop a desire for caring among the students:

*Before I met the patient, I felt lazy to start it up [give intervention], but once I started [the interaction], I want to give, and want to give again. […] I used the same approach, just starting to give health education. When the client gave a positive response, then I felt good (Student 1 NC1)*.

The above quote highlights that students’ capacity for caring was developed through providing care for families and people in the community. The students’ interactions with the clients were useful in developing a desire for caring that can lead to the development of students’ caring behaviours. The NC model is designed to provide a consistent caring environment for students and nurses. Students who have learnt about caring for patients in the classroom can then apply this knowledge consistently in the field.

### Nursing education using a service learning approach

In this section, pattern matching analysis was used to analyse the documents and interview data in order to compare this data to the pattern of possible explanations. Analysis of the first publication of the Indonesian NC (Samba, 2007; 2012) showed that the theoretical basis for the Indonesian NC consisted of six concepts: CHN services as a system, adult learning, professional organisation, caring, nursing research, and community. This finding was different from the interview findings where lecturers identified three roles associated with the NC, being education, research, and community service, summarised in the following quote:

*The concepts of the nursing centre are education, in which students take part in community placements; research, in which academics and students conduct research; and community service, in which the academic gives service to the community (Lecturer 2 from NC1)*.

In the document, Samba (2012) also proposed both adult learning and active learning as the learning approaches in the NC. This may have contributed to the confusion of students in applying the appropriate learning approach, as indicated by the following student statement:

*The truth is that the placement in the NC is the most unclear work I have ever had (Student 1)*

The data suggest that students from the three NC sites were confused about their roles in the NC, which then appears to contribute to the inadequate functionality of the NCs. The findings from the document analysis showed that an effective and active learning approach was the intention of the NC founder:

*In order to learn effectively, nursing students need a learning method that is active, integrative, cumulative, and consistent. Active learning demands creativity, independent thinking, collaboration, and learning directed by the student. Integrative learning is needed in order to give real benefit to the community (Samba, 2012, p.19)*.

The concepts of active, integrative, cumulative and consistent learning do not belong exclusively to the concept of adult learning (Merriam and Bierema, 2013), but are also embodied in the concepts of active learning (Dewing, 2008) and service learning (Whiteet al., 1999). In order to identify an educational method that can facilitate integration at an optimal level in the NC, the following Table 3 presents an overview of the pattern matching analysis using the three concepts mentioned above.

**Table 3.** Pattern matching analysis of the learning approach in the NC

Learning approach and components of learning	Data examples	Researcher interpretation of learners	Match to the NC characteristics
Adult Learning (Merriam & Bierema 2013)			
Students’ self-concept moves from a dependent personality towards a self-directed one	*I think placement in the community is too relaxed and less motivating for the future. We should be given a clearer target (Student 2 NC3)*	Dependent	No
Experience as a cumulative resource for learning	*We [students] focus on practice in the family and the community (Student 2 NC2)*	Focus on practice experience	Yes
Orientation to learning shifts from one of subject-centeredness to one of problem-centeredness.	*We have courses of 6-2 application for family, community, and geriatric, so students must have a placement in the NC during these courses (Lecturer 2 NC3)*	Subject/task centeredness	No
Internal motivation to learn	*They [students] do not want to do their tasks, difficult to complete the task (Head of Puskesmas 3)*	No internal motivation	No
Readiness to learn	*I felt that collaboration with the students was difficult because the students tend to be too passive (Nurse 3 NC1*)	Some students are not ready to learn	No
Active Learning (Dewing 2008)			
Based in, and on, personal work experience of practitioners	*Lecturer must give us clear guidance. Even though I have previous work experience in the hospital, I was confused about what to do in the NC (Student 3 NC1)*	Students’ personal work experience might not be applicable in the NC	No
Dialogue with self	There is no data to show this	Dialogue with self is not evident in the data	No
Observing	*I did not give health education alone; sometimes with a nurse and sometimes with my fellow students (Student 1 NC1)*	Students do not observe, instead, they are doing the activities	No
Dialogue with others. One-to-one or group dialogue between practitioners about a practice topic or activity	*We also do monthly discussions and reflection on cases with the Head of the Puskesmas (Nurse 1 NC3)*	Dialogue and reflection are used by health service stakeholders	Yes
Doing (case studies, role-playing, simulation activities, and other activities)	*All students must provide family nursing care for families in the community (Lecturer 1 NC2)*	Students provide services in the real-life setting	Yes
Service learning (White *et al*. 1999)			
A course-based credit-bearing educational experience	*I have got a new experience because of this CHN placement in the city, we know about diseases in the community and we learn about epidemiology too (Student 1 NC3)*	Students learn from the experience	Yes
Reflect on the service activity	*All students have to write a journal while they are on placement, they must write their everyday learning activities (Lecturer 3 NC1)*.	Students’ reflective activities through journal writing	Yes
Participate in an organised service activity that meets identified community needs	*I know that the benefits [of the NC] are very big, especially for the data (Provincial Coordinator of the CHN program)*	Reciprocal learning for stakeholders	Yes
To gain further understanding of course content, a broader appreciation of the discipline and an enhanced sense of civic responsibility.	*For my future career as a nurse, I think the communication skills with people in the community could be useful and applied everywhere (Student 2 NC1)*.	Students gained further understanding of course content and its application for future career.	Yes

While there were confusion in the practice and overlapping components of the adult, active and service learning evident in the data, all components of service learning are evident and matched the characteristics of the NC model in West Java, Indonesia.

### The NC as a model for invigorating community health nursing services

In addition to educational purposes, the NCs were also used as a venue for invigorating CHN in West Java, Indonesia “*because CHN practice did not exist. […] an idea of NC is to revitalise the Community Health Nursing program” (Suharyati Samba, the founder of NC Indonesia)*. The term ‘Nursing Centre’ is used by the founder of the NC and the Provincial Health Office as a strategy to give new meaning, new energy and new activities to CHN practice in Indonesia. Participants from the community health centres, including the Head of the *Puskesmas* and the nurses, viewed the CHN program, known as *Perkesmas (Perawatan Kesehatan Masyarakat)* as the basis of the NC model. The NC was perceived as a venue to conduct the Perkesmas program, as stated by a head of Puskesmas:

*I think the NC is a place for nurses to conduct Perkesmas programs inside the Puskesmas building. Nurses’ outreach activities are organised by the person-in-charge of the Perkesmas programs (Head of Puskesmas 2)*.

Through a CHN (*Perkesmas)* program that was integrated into the NC model, nurses had the opportunity to contribute to the wider community:

*Through the NC, we have an opportunity to do the follow-up; nurses will assess and determine the priority of patients who need home visits; from there, we can follow-up and the intervention is not only for one patient but also for a whole family (Head of Puskesmas 3)*.

The CHN nurses who worked through the NC had the potential to deliver a holistic form of care, which then had a positive impact on the community. The necessity of holistic activities taking place both inside the facilities and through outreach activities as ways of providing care is also found in the NC document analysis (Samba, 2002; 2012). Hence, the role of the NC is not merely to fulfil nursing education or lecturers’ needs, but also to provide a holistic form of nursing care for individuals, families, and communities.

### Service improvement through research and community service activities

The NC was seen as a way of facilitating the three roles of education, research and community service in Indonesian higher education as per the following participant example:

*The lecturers’ roles in the NC are related to three obligations of higher education [in Indonesia]. We need to provide services for the students and to anyone who requires the service. Besides education, we also have to do research and community services (Lecturer 2 NC2)*.

The importance of research was also expressed by the Heads of *Puskesmas* to improve services in the NC and the *Puskesmas*:

*I always welcome students and lecturers who want to do research here. It is very important to improve the Puskesmas performance. We [Puskesmas staff] do not have time to do research, so I always ask the students to do research here and give us the reports (Head of Puskesmas 3)*.

These findings show that the lecturers, nurses, and the Heads of the *Puskesmas* recognise the importance of research in the NC. However, participants stated that the research activities by students and staff could not be conducted optimally due to the time constraints on both lecturers and community health centre staff.

The NCs were also used as community service sites for lecturers as stated by one of the lecturers from NC2:

*All the lecturers here want to work in the NC as part of their community service activities. I arrange the schedule for the lecturers to work in the NC. Most of the activities are health education and counselling in the NC, but now we have added home visits and school health activities (Lecturer 3 NC2)*.

Research and community service activities would reduce the gap between CHN services and education through the process of knowledge-sharing towards best practice in the Community Health Centres. Knowledge-sharing that is related to evidence-based practice and research would also increase the likelihood of these best practices being applied in the NC by students and nurses. Best practice will also increase the quality of CHN service provision for individuals, families, and people in the community, which has the potential to produce better health outcomes overall.

## Discussion

**Figure 1.**
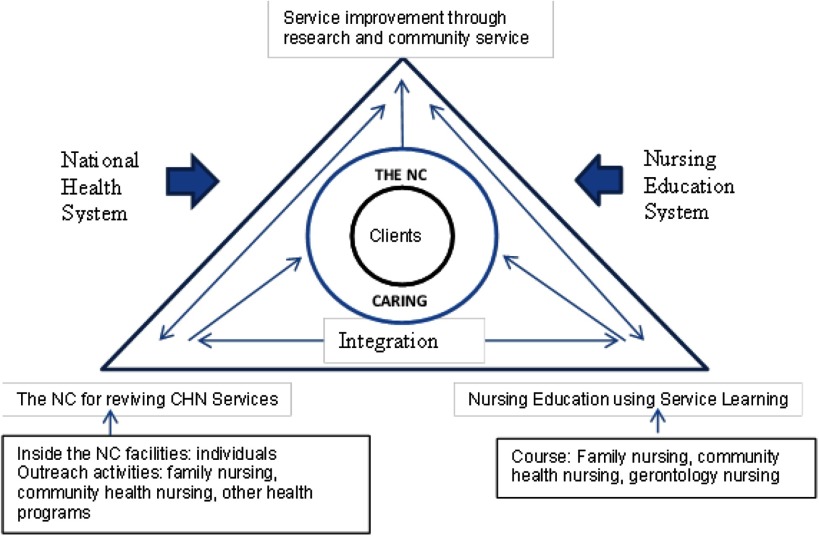
Conceptual framework for the integration in the nursing centre

The components identified in the findings section were used to construct a conceptual framework of the NC as shown in Figure 1. These components form a triangle of integration of CHN services, education, and research with client-centred care as the core of the framework in order to clarify the relationships between these three components of the NC that shows the tri-partite relationships between CHN services, education, and research in the NC model. CHN services and education using service learning are at the bottom of the triangle because these are the fundamental activities that support research and community service activities,which are placed at the tip of the triangle. CHN services consist of inside the NC facility as well as outreach activities. The identification of health problems and community needs during the integration of CHN services and education could become a topic of further research and community service activity.

Figure 1 shows the interrelationships between the components in the NC model. The integration of nursing services and education in the NC could contribute to service improvement through research and community service activities. The results of research and community service would inform further improvement of nursing services and education. The arrow directions mean that each of the components influences each other. If the integration of CHN services and education improved, then it would likely induce collaboration and partnerships needed to perform the research and the community service program so that new knowledge and innovation can be produced. In a circuitous way, this knowledge and innovation would likely lead back to the improvement of CHN services and education within the NC.

In this conceptual framework, the clients, who consist of individuals, families, and the community, are the core of the NC model as the shared common ground for both nursing education and health service stakeholders to integrate CHN services, education, and research. Shared mission, values, goals, and measurable outcomes are important to maintain a collaborative approach (Berkowitz, 2000). In this way, all stakeholders can actively and jointly establish roles, norms, and processes based on contributions from, and the shared common ground of all stakeholders. This shared common ground would help to facilitate effective interaction among the stakeholders and would ultimately produce better outcomes (Foss *et al*., 2003). A shared common ground is crucial for inter-sectoral collaboration to strengthen primary and community care, as well as to better coordinate care around people’s needs (Walley *et al*., 2008). This could lead to increased access to comprehensive and high-quality health services (WHO, 2015).

This proposed conceptual framework is different from other NC models as it incorporates a service learning approach and the integration of CHN practice, education, and research in the model. The findings show that by using service learning in the NC model, nursing students could learn from the experience of working collaboratively with the community, nurses and other health professionals. Service learning in this study is defined as a structured form of intra-curricular experiential learning that engages students in service and learning through real-life experiences, using reflection and reciprocity as tools to achieve the specified outcomes and benefits for all stakeholders (Juniarti *et al*., 2016). This conceptual framework is different from the original six concepts proposed by Samba (2002) for the Indonesian NC model. The original concepts did neither identify a clear shared common ground between the stakeholders nor clarify the responsibilities and roles of nursing education and health service stakeholders. It is argued that service learning is an appropriate educational approach for the NC because this approach is able to support a clear shared common ground and clarify responsibilities and roles between the stakeholders to support collaborative project and reciprocal benefits (Berry and Chrisholm, 1999; Voss *et al*., 2015). This current study shows that service learning is an appropriate and potentially productive learning approach in the NC. This is similar to the findings from other studies (Connolly *et al*., 2004; Lough, 1999; Lutz *et al*., 2001; Marek *et al*., 2004; Yeh *et al*., 2009) which report service learning as a specific approach to student education in the NC alongside service provision for clients and research.

The conceptual framework of the NC is developed for the purposes of reviving CHN services. Through the NC, nurses are encouraged to integrate a range of programs using the CHN approach as well as to conduct family nursing activities. Involving the key stakeholders in collaborative research activities could become a strategy to build on the desire and willingness of the champions in the nursing education institution and Community Health Centre to improve the quality of community nursing care and students’ education in the NC. However, research agendas in the NCs need to be aligned with the needs of people in the community using participatory recruitment strategies and multiple data collection methods to build reciprocity and maintain trusting relationships with the community (Zachariah and Lundeen, 1997). Such trusting relationships need to be maintained over the long term to ensure the sustainability of the NC (King, 2008). Integrating CHN services, education, and research would overcome the problem of the sustainability of the NC because it helps to build trusting relationships between stakeholders.

This NC framework shows the clear roles of both the nursing education and the health service stakeholders. The role of the nursing education stakeholders is to provide service learning, research, and community service, while the role of the health service stakeholders is to provide family nursing services, CHN services, and other health programs. The roles of these two organisations are integrated through a collaborative approach which is co-located in the NC.

In the NC, students learn to deliver CHN and family nursing care in the *Puskesmas* coverage area through partnerships and collaboration with the NC stakeholders. The clients receive services in a caring environment. Through interacting with clients, students could develop a sense of caring for their clients in the community as well as in the hospitals when they graduate. These insights into caring develop when students and nurses engage directly with patients, families, and the community (Morse *et al*., 2006). Caring behaviours also develop when the conditions within the placement setting are supportive of students (Sikma, 2006). Students, nurses, and lecturers who work together in the NC engage with clients at the individual, family, and community levels. Through these forms of engagement, students not only learn about the topic matter, but also enhance their understanding of the meaning of being a nurse, a citizen, and a member of the community (Seifer and Vaughn, 2002).

## Conclusion

The findings from this study have contributed to a new understanding of the components of integration in the NCmodel. These components are: (1) client-centred care as the shared common ground for integration in the NC; (2) nursing education using a service learning approach; (3) the NC as a model for reviving CHN services; and (4) service improvement through research and community service activities. In this framework, there is a link between nursing education and the community health centre stakeholders through integrated care and service learning that enables greater collaboration and integration in the NC. This greater collaboration and integration in the NC will, in turn, lead to the more effective functioning of the NCs. It is argued that all of these components need to operate in a conducive environment within the NC in order to develop the caring insights of nurses and students who are involved in the NC.

This study shows that the service learning approach is suitable for a model that integrates education and health service institutions because it links the services with the students’ learning process. The components that have been reported in this paper can be used as a conceptual framework with clarified relationships between each component as the basis for the development of future evaluation plans for the NC. The implications of this work have the potential to support policies that will improve the health of individuals and communities through the integration of student education, community health service delivery, and research. This study has added new knowledge to the NC and service learning fields by providing a clear understanding of the conceptual framework of the NC as a ‘blueprint’ for the integration of CHN services, education, and research. The proposed framework would provide clarity for other researchers who would like to apply this model to address a specific health issue in the community. The application of the NC model to address specific health issues in the community would strengthen the framework and increase its applicability in different community settings in Indonesia and across the globe. However, there is a need for further research that encompasses the applicability and suitability of the framework in other NC settings
